# Neoadjuvant Chemotherapy for T3 Tumors in the Era of Precision Medicine—Biology Is Still King

**DOI:** 10.3390/ijms26020491

**Published:** 2025-01-09

**Authors:** Rakhshanda Layeequr Rahman, Alfredo Santillan, Mehran Habibi, Peter Beitsch, Pat Whitworth, Harshini Ramaswamy, Nicole Chmielewski-Stivers, Andrea Menicucci, William Audeh, Joyce O’Shaughnessy

**Affiliations:** 1 School of Medicine, Texas Tech University Health Sciences Center, Lubbock, TX 79430, USA; 2 Texas Oncology, San Antonio, TX 78240, USA; 3 Northwell Health, Staten Island, NY 10305, USA; 4 Dallas Surgical Group, Dallas, TX 75231, USA; 5 Nashville Breast Center, Nashville, TN 37203, USA; 6 Agendia Inc., Irvine, CA 92618, USAwilliam.audeh@agendia.com (W.A.); 7 Baylor University Medical Center, Texas Oncology, Dallas, TX 75246, USA

**Keywords:** cT3, locally advanced disease, neoadjuvant chemotherapy (NAC), pathological Complete Response (pCR), 70-gene signature, 80-gene signature, molecular subtyping, precision oncology

## Abstract

Clinical T3 (cT3) breast cancer (BC) presents a challenge for achieving cosmetically acceptable breast conservation, and neoadjuvant chemotherapy (NAC) is commonly used for cytoreduction in these high-risk cancers. MammaPrint^®^ risk-of-recurrence and BluePrint^®^ molecular subtyping genomic signatures have demonstrated high accuracy in predicting chemotherapy benefits. Here, we examined the utility of MammaPrint/BluePrint for predicting pathological Complete Response (pCR) rates to NAC among 404 patients diagnosed with cT3 early-stage BC. The association of genomic subtype and clinical features with the likelihood of pCR was evaluated by multivariate logistic regression. Differences in pCR rates between genomic risk categories were evaluated by a two-sided proportional z-test and stratified by nodal status. MammaPrint/BluePrint subtyping was associated with significantly higher odds ratios (ORs) for pCR in MammaPrint High-Risk/BluePrint Basal-Type (OR = 3.06, 95% CI: 1.15–8.19, *p* = 0.025) and HER2-Type (OR = 6.27, 95% CI: 2.19–19.38, *p* = 0.001) compared to BluePrint Luminal-Type. Of the 209 patients with hormone receptor-positive, HER2-negative disease, 6.7% achieved pCR, and MammaPrint High-Risk was associated with a significantly higher pCR rate (9.3%) compared to MammaPrint Low-Risk cancers (0%), regardless of nodal involvement (*p* = 0.036). These data show that for patients with MammaPrint Low-Risk, cT3 tumors are less likely to have clinically impactful cytoreduction from NAC, regardless of nodal involvement.

## 1. Introduction

Clinical T3 (cT3) breast cancer, particularly node positive disease, continues to be a vexing problem for clinicians, because the large tumor size may render upfront surgical resection challenging and breast-conserving surgery untenable. For this reason, major cancer societies, such as the American Society of Clinical Oncology (ASCO) [1] and the National Comprehensive Cancer Network (NCCN) [2], recommend neoadjuvant chemotherapy therapy (NAC) for down-staging for cT3 cancers. The goal of NAC in T3 breast cancer is to effect resectability and to facilitate cosmetically acceptable breast conservation. In patients with lymph node-positive (N+) disease, an added advantage is the potential for conversion to node-negative (ypN0) status in approximately 40% of patients, thus allowing for less axillary surgery [3]. However, it remains a major clinical dilemma to understand which patients with cT3 cancers, especially HR+HER2− cancers, will benefit from NAC with substantial enough reduction in tumor burden to allow for breast conservation and/or less axillary surgery.

The last decade has witnessed significant progress in delineating the genomic diversity of breast cancer. This progress has provided new insights into not only the natural history of breast cancer, but also into identifying unique cohorts of patients that benefit from chemotherapy and targeted therapies [4]. The incorporation of genomic profiles into the clinical assessment of breast cancer has led to the delineation of unique breast cancer subgroups with distinct outcomes and vulnerabilities to targeted therapies [5]. This research has redefined the diagnostic landscape of breast cancer, revealing novel molecular subgroups with distinct clinical outcomes and subtype-specific putative driver genes. This paradigm shift provides unique insights and opportunities for more precise treatment decisions, reducing the risk–benefit ratio of toxic therapies compared to relying on anatomical staging alone. Genomic subtyping also offers an opportunity to enhance the efficacy of the Choosing Wisely (CW)^®^ campaign, which was initiated by the American Board of Internal Medicine to encourage patient–physician conversations about unnecessary interventions [6]. The CW campaign has led to the de-escalation of therapies that are unlikely to contribute to enhanced survival or quality of life, particularly in the disciplines of surgery and radiation [7]. Notably, ASCO guidelines maintain that there is insufficient evidence to support the use of genomic profiles for decision making regarding the clinical utility of NAC for locally advanced breast cancer [1].

In order to delineate the role of genomic profiling in predicting response to NAC in locally advanced breast cancer, the current study examined response to NAC in cT3 breast cancers that were genomically profiled by MammaPrint^®^ (70-gene assay) and BluePrint^®^ (80-gene assay) in an analysis of prospectively collected, pooled data from the NBRST [8], MINT [9], and FLEX [10] clinical trials. A tumor biology assessment through genomic profiling of cT3 cancers may provide useful information about which patients with locally advanced cancers respond well to NAC, as genomic profiling does predict for systemic benefit from neo/adjuvant chemotherapy.

## 2. Results

A total of 404 patients (214 from NBRST, 123 from FLEX, 67 from MINT) met eligibility criteria. Baseline characteristics are depicted in Table 1. The median (SD) age was 52 (±12) years, and more than half (51.7%) of the patients were premenopausal. Two hundred and ninety-five (73.1%) patients had node-positive disease, and most patients (53.2%) had Grade 3 tumors. Sixty-five patients (16.1%) had MammaPrint Low-Risk tumors, while 329 (83.9%) patients had MammaPrint High-Risk tumors. The majority of patients (51.7%) were diagnosed with HR+HER2− disease.

**Figure 1:**
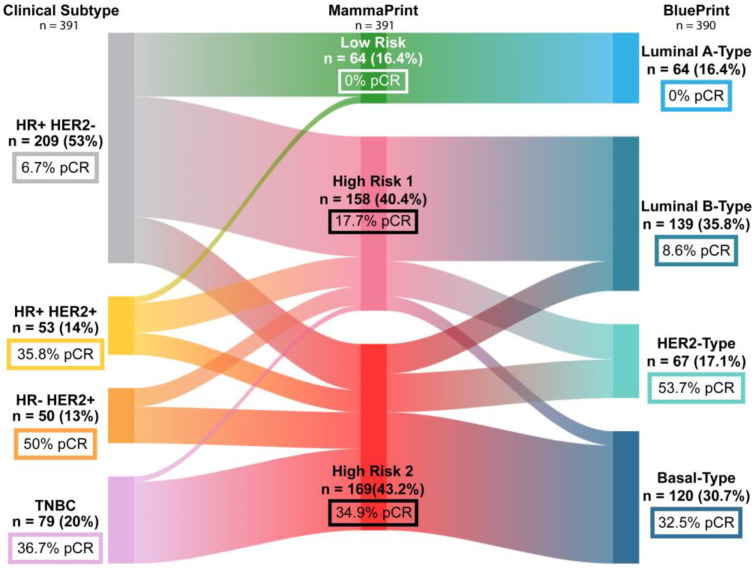
Pathological Complete Response (pCR) rates by clinical and molecular subtyping. Sankey diagram depicting reclassification of clinical subtypes of all patients with cT3 tumors to MammaPrint and BluePrint genomic subtyping. Patients with unknown receptor status (*n* = 13) and not-requested BluePrint (*n* = 1) were excluded.

Overall, 87 of 404 patients had no residual tumor on final pathology for a pCR rate of 21.5%. Figure 1 illustrates the strength of prediction of pCR by receptor status versus MammaPrint and BluePrint molecular profiling. Traditional biological marker profiles predicted the highest rate of pCR among patients with HR−HER2+ (50%, *n* = 50), and the lowest pCR rate with HR+HER2− (6.7%, *n* = 209). Among the 209 patients with HR+HER2− disease, 61 were classified with MammaPrint Low-Risk tumors. Among all patients, no (0%) MammaPrint Low-Risk tumors (*n* = 65) achieved pCR, compared to a 16.8% and 34.3% pCR rate for High-Risk 1 and High-Risk 2 tumors, respectively (*p* = 0.0002).

To explore the role of molecular profiling, logistic regression analysis was performed to evaluate the prediction of pCR, with clinical and phenotypic profiles as confounders included in the model in addition to the BluePrint molecular profile (MammaPrint was excluded due to zero pCR in Low-Risk group) (Table 2). Logistic regression revealed that BluePrint subtyping showed significantly higher odds ratios for pCR in Basal-Type (OR = 3.06, 95% CI: 1.15–8.19, *p* = 0.025) and HER2-Type (OR = 6.27, 95% CI: 2.19–19.38, *p* = 0.001) compared to the reference category (Luminal-Type). It is important to note that HR+HER2+ disease designated through conventional testing is associated with higher odds of pCR (OR = 2.91, 95% CI: 0.97–8.23, *p* = 0.048); however, the association was stronger, with a molecular profile of HER2-amplified disease (OR = 6.27, 95% CI: 2.19–19.38, *p* = 0.025), suggesting heterogenous biological behavior within HER2+ disease. Most importantly, traditional high-risk factors utilized as indicators for chemotherapy, such as menopausal status, nodal status, and grade, were not associated with pCR.

**Figure 2:**
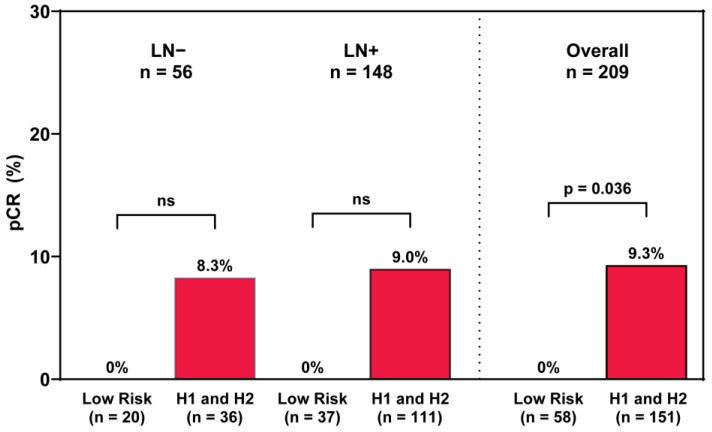
pCR rates by nodal status and MammaPrint for patients with HR+HER2− disease. Differences in pCR rates were evaluated by two-sided proportional z-tests and stratified by nodal status, with *p* < 0.05 indicating statistical significance. Abbreviations: H1, High-Risk 1; H2, High-Risk 2.

Because those with HR+HER2− tumors, which are less likely to respond to NAC, were offered NAC based on anatomical staging, this group was further examined to assess the association between pCR and molecular profiling. Of the 209 (51.7%) patients with HR+HER2− disease, 6.7% (14) achieved pCR; MammaPrint/BluePrint Low-Risk and Luminal A tumors (*n* = 58) among this group had 0% pCR, irrespective of nodal involvement at presentation (*n* = 37 LN+, MammaPrint Low-Risk) (Figure 2). Within this clinical group, patients with MammaPrint High-Risk disease (*n* = 151) had a pCR rate of 9.3% (*p* = 0.036). By BluePrint molecular subtype, pCR was achieved for 7 (5.8%) of the 120 Luminal B, and 7 (23.3%) of the 30 Basal-Type tumors.

## 3. Discussion

Genomic profiling has emerged as an important mainstay in treatment planning for early-stage HR+HER2− breast cancer. The appropriate adoption of genomic testing in clinical care improves quality of life by allowing the for omission of adjuvant chemotherapy in patients with genomically low-risk disease, regardless of larger tumor size and nodal status. Still, one challenge of genomically low-risk, locally advanced disease remains that clinicians recommend NAC based on high-risk anatomic features, knowing that these low-risk cancers do not have improved disease-free survival with adjuvant chemotherapy [11,12,13,14].

This study describes the likelihood that patients with locally advanced disease would develop a pCR with NAC according to their genomic risk, assessed by MammaPrint, as well as their biologic subtype, assessed by BluePrint. Regardless of which of the available genomic assays is used to assess risk and tumor biology, it has been known for over two decades that Luminal A breast cancers are unlikely to benefit from chemotherapy with regard to improved disease-free survival [15]. Glück et al. [16] analyzed data from 437 patients from four NAC trials and reported that only 6% of patients in the Luminal A-Type group, as determined by the MammaPrint/BluePrint profiling, had pCR. Furthermore, the 5-year distant metastasis-free survival in this Luminal A group (*n* = 90; which included seven HER2+ and eight triple-negative breast cancer [TNBC] patients) was 93%. The Neoadjuvant Breast Registry Symphony Trial (NBRST) compared IHC-based markers and MammaPrint/BluePrint genomic assays in predicting for pCR and demonstrated that only 2% of patients in the Luminal A group experienced pCR [17]. In contrast, MammaPrint/BluePrint genomically High-Risk Luminal B and Basal tumors were more likely to achieve a pCR in this study. A recent whole transcriptome analysis from ISPY2 found significant correlation between HR+HER2− MammaPrint High-Risk tumors and TNBC. These findings suggest that genomically High-Risk tumors have a similar biology to TNBC and are candidates for receiving chemotherapy [18].

In the present study, 24.4% of HR+HER2− patients treated with NAC were found to have BluePrint Luminal A-Type disease on genomic profiling. Lannin et al. [19] queried the Surveillance, Epidemiology, and End Results (SEER) database, analyzing the relationship of tumor size with tumor biology in invasive breast cancers diagnosed in 2001–2013. They reported that both tumor size and biologic features impact prognosis, noting that patients with large, biologically favorable cancers can have a better prognosis than those with small, biologically high-risk cancer. They also observed that the difference in prognosis according to tumor size is smaller for biologically favorable cancers and greater in those with biologically unfavorable disease.

There is a growing consensus among oncologists that patients with HR+ HER2− Luminal A breast cancer generally do not benefit from adjuvant chemotherapy. However, there is a lack of consensus regarding the clinical utility of incorporating genomic profiling into decision making about which locally advanced HR+ HER2− patients should receive NAC. An IHC-based assessment of hormone receptors, proliferative index, and grade are routinely used to understand patients’ prognosis and the sensitivity of their cancers to cytotoxic and hormonal therapies. However, genomic subtyping and IHC-based biological assessments provide discordant results in up to 30% of early breast cancers [20]. Prat et al. [21] reviewed the concordance between surrogate IHC-based and PAM50-based intrinsic subtyping and found a discordance rate of 30.7%; only 62% of HR+ HER2− tumor samples that were IHC-based Luminal A cancers were genomically classified as Luminal A, while 34% of patients with IHC-based Luminal B tumors were genomically classified as Luminal A. Whitworth et al. [17] and Yao et al. [22] assessed the concordance of MammaPrint/BluePrint signatures with IHC-determined molecular subtypes and found a 22–25% discordance rate.

An important question is the relevance of utilizing pCR as an outcome measure for making clinical decisions for MammaPrint/BluePrint Luminal A-Type cancers. Symmons et al. [23] reported on the association of long-term prognosis with a residual cancer burden index for each tumor subtype based on IHC/FISH classification. They reported that HR+HER2− cancers were associated with 10% pCR rate, 13% RCB class I, 60% RCB class II, and 17% class III. Extensive residual disease imparted significantly worse prognosis; however, in the multivariate model for recurrence-free survival, RCB index (HR = 2.28; 95% CI, 1.76 to 2.96), pretreatment clinical stage III (HR = 2.51; 95% CI, 1.71 to 3.69), and pCR (hazard ratio, 5.03; 95% CI, 1.60 to 15.78) were independently prognostic, whereas age, grade, and multifocality were not. A limitation of this current study is the lack of information on the RCB class from the NBRST, FLEX, and MINT trials. The FLEX registry is now collecting RCB class data, which will allow for future analyses to incorporate this variable as well as disease-free survival outcomes in long-term follow up for patients with T3 HR+HER2− disease.

The present study demonstrated the utility of MammaPrint/BluePrint testing in predicting pCR to NAC, regardless of tumor staging. Whereas 137/209 (65.5%) of all HR+HER2- tumors were considered High-Risk by MammaPrint and were more likely to have a pCR with NAC, no HR+HER2− MammaPrint Low-Risk tumor had a pCR with NAC. A limitation of this study is that retrospective nature of this analysis, though the data were collected prospectively. This can potentially be associated with selection bias; however, our confounder analysis shows that the tumor size is the most likely bias that led to the use of NAC in these patients. Another limitation is that the study reports on pCR but not on partial response data that potentially convert some mastectomy candidates to breast conservation. Ultimately, long-term outcome data may demonstrate that these MammaPrint Low-Risk patients are better candidates for neoadjuvant endocrine therapy (NET). Van Olmen et al. [24] reported on 72 patients treated with neoadjuvant endocrine therapy, 54% of whom were able to have breast conservation after an initial assessment of requiring mastectomy. More data are needed regarding the utility of MammaPrint/BluePrint for predicting NET response.

## 4. Materials and Methods

### 4.1. Patient Cohort

This study included patients with cT3 cancers enrolled in NBRST (NCT01479101), FLEX (NCT03053193), and MINT (NCT01501487) trials who had MammaPrint and BluePrint profiling from core biopsy specimens. All patients received NAC followed by surgical resection and had post-surgical pathological Complete Response (pCR) data. Demographic variables included age, race, and menopausal status; oncologic variables included tumor phenotype based on histology, hormone receptor (HR), human epidermal growth factor (HER2) receptor status, tumor grade, and nodal status.

### 4.2. Molecular Subtyping

Tumors were classified using the MammaPrint 70-gene risk-of-recurrence signature as having a Low-Risk (index value > 0) or a High-Risk (index value ≤ 0) of distant recurrence [11,25]. MammaPrint High-Risk tumors were further stratified into High-Risk 1 (index value ≤ 0 to ≥−0.570) or High-Risk 2 (index value < −0.570 to −1) subgroups [26,27]. The BluePrint 80-gene molecular subtyping signature identified tumors as Luminal-Type, HER2-Type, or Basal-Type [28]. Together, MammaPrint and BluePrint further distinguished Luminal-Type tumors as Low-Risk, Luminal A-Type or High-Risk, Luminal B-Type tumors.

### 4.3. Statistical Analysis

Pathological Complete Response was identified as the main outcome measure. Molecular profile was identified as an independent variable, and age, race, menopausal status, tumor type, and nodal status were identified as confounding variables for analysis. Descriptive statistics were calculated to summarize the clinical characteristics of the study population. Univariate analyses were performed using Chi-squared or Fisher’s Exact Test to compare categorical variables, while *t*-tests or Mann–Whitney U tests were applied for continuous variables as appropriate. Differences in pCR rates between genomic risk categories were evaluated by two-sided proportional z-test and stratified by nodal status. The association of genomic subtype and clinical features with the likelihood of pCR was evaluated by multivariate logistic regression. Variable selection was guided by clinical relevance, and multicollinearity was assessed using variance inflation factors (VIFs). The model was evaluated for goodness of fit using the Hosmer–Lemeshow test. Statistical significance was set at a two-sided *p*-value < 0.05, and all analyses were performed in R (version 4.1.1) and SAS (version 9.4).

## Figures and Tables

**Table 1:** Baseline clinical characteristics of patients with cT3 tumors.

Clinical Characteristics	No. Patients (%) (*n* = 404)
Age in years—Mean (SD)	52 (±12)
Menopausal Status	
Pre	186 (51.7)
Post	209 (46.04)
Unknown	9 (2.2)
Race	
White	293 (72.5)
Black	67 (16.6)
Latin/Hispanic	24 (5.9)
AAPI	12 (2.97)
Other	3 (0.7)
Unknown	5 (1.2)
Histopatholgical Type	
IDC	325 (80.5)
ILC	49 (12.1)
Mixed IDC/ILC	18 (4.5)
Other	10 (2.5)
Unknown	2 (0.5)
Nodal Status	
N0	104 (25.7)
N1	237 (58.7)
N2	38 (9.4)
N3	12 (3.0)
NX	8 (2.0)
Unknown	5 (1.2)
Grade	
G1	20 (5.0)
G2	150 (37.1)
G3	215 (53.2)
GX	13 (3.2)
Unknown	6 (1.5)
Receptor Status	
HR+HER2−	209 (51.7)
HR+HER2+	53 (13.1)
HR−HER2+	50 (12.4)
TNBC	79 (19.6)
Unknown	13 (3.2)
MammaPrint	
Low Risk	65 (16.1)
High Risk 1	167 (41.3)
High Risk 2	172 (42.6)
BluePrint	
Luminal A-Type	64 (15.8)
Luminal B-Type	150 (37.1)
HER2-Type	68 (16.8)
Basal-Type	121 (30.1)
Not Requested	1 (0.3)

Data represented as n (%), unless otherwise specified. Abbreviations: n, number of participants; SD, standard deviation; AAPI, Asian, Asian American, or Pacific Islander; IDC, Invasive ductal carcinoma; ILC, Invasive lobular carcinoma; NX, lymph node status; HR, hormone receptor; HER2, human epidermal growth factor receptor 2; TNBC, triple-negative breast cancer.

**Table 2:** Multivariate analysis of predictive factors for pathological Complete Response (pCR).

Characteristic	Odds Ratio	95% CI	*p*-Value
BluePrint Subtype			
Luminal (*n* = 214)	1		
Basal (*n* = 121)	3.06	[1.15, 8.19]	0.025
HER2 (*n* = 68)	6.27	[2.19, 19.38]	0.001
Menopausal Status			
Pre/Peri (*n* = 186)	1		
Post (*n* = 209)	0.66	[0.36, 1.19]	0.173
Receptor Status			
HR+HER2− (*n* = 209)	1		
HR+HER2+ (*n* = 53)	2.91	[0.97, 8.23]	0.048
HR−HER2+ (*n* = 50)	2.59	[0.82, 8.05]	0.101
TNBC (*n* = 79)	2.33	[0.91, 6.34]	0.085
Lymph Node Stage			
LN− (*n* = 104)	1		
LN+ (*n* = 287)	1.08	[0.55, 2.18]	0.816
Grade			
G1 (*n* = 20)	1		
G2 (*n* = 150)	2.77	[0.39, 56.98]	0.38
G3 (*n* = 215)	4.49	[0.66, 91.11]	0.191

Data represented as OR (95% CI, *p*-value). *p* < 0.05 indicates significant risk factor. Abbreviations: OR, odds ratio; CI, confidence interval.

## Data Availability

The clinical datasets produced and/or analyzed in this study can be obtained from the corresponding author upon reasonable request.
